# Utility of the Plasma Level of suPAR in Monitoring Risk of Mortality during TB Treatment

**DOI:** 10.1371/journal.pone.0043933

**Published:** 2012-08-28

**Authors:** Paulo Rabna, Andreas Andersen, Christian Wejse, Ines Oliveira, Victor Francisco Gomes, Maya Bonde Haaland, Peter Aaby, Jesper Eugen-Olsen

**Affiliations:** 1 Bandim Health Project, Indepth Network, Bissau, Guinea-Bissau; 2 Department of Infectious Diseases, Aarhus University Hospital, Aarhus, Denmark; 3 Center of Global Health, School of Public Health, Aarhus University, Aarhus, Denmark; 4 Clinical Research Centre, Copenhagen University Hospital Hvidovre, Hvidovre, Denmark; 5 Bandim Health Project, Statens Serum Institute, Copenhagen, Denmark; Fundació Institut d’Investigació en Ciències de la Salut Germans Trias i Pujol. Universitat Autònoma de Barcelona. CIBERES, Spain

## Abstract

**Objective:**

To investigate whether changes in the plasma level of soluble urokinase plasminogen activator receptor (suPAR) can be used to monitor tuberculosis (TB) treatment efficacy.

**Design:**

This prospective cohort study included 278 patients diagnosed with active pulmonary TB and followed throughout the 8-month treatment period.

**Results:**

Mortality during treatment was higher in the highest inclusion quartile of suPAR (23%) compared to the lowest three quartiles (7%), the risk ratio being 3.1 (95% CI 1.65–6.07). No association between early smear conversion and subsequent mortality or inclusion suPAR was observed. After 1 and 2 months of treatment, an increase in suPAR compared to at diagnosis was associated with a Mortality Rate Ratio (MRR) of 4.5 (95%CI: 1.45–14.1) and 2.1 (95%CI 0.62–6.82), respectively, for the remaining treatment period.

**Conclusions:**

The present study confirmed that elevated suPAR level at time of initiation of TB treatment is associated with increased risk of mortality. Furthermore, increased suPAR levels after one month of treatment was associated with increased risk of mortality during the remaining 7-month treatment period.

## Introduction

The progression of the tuberculosis (TB) epidemic, especially in areas of HIV infection, requires urgent and effective action. Despite substantial progress in the past decade, TB remains a major public health problem in most parts of the world [1]–[3]. The incidence of acute tuberculosis is high in many countries, and failure to diagnose and treat TB at an early stage may result in serious complications. A community study in Guinea-Bissau estimated the incidence of tuberculosis to 471 per 100.000 persons-years [4].

In resource-limited settings, efficacy of pulmonary tuberculosis treatment is assessed mainly by the disappearance of mycobacterium from sputum. Culture is time-consuming, costly and there is delay in getting the results. Moreover, appropriate facilities are not universally available especially in low-income countries.

Urokinase-type plasminogen activator receptor (uPAR) is predominantly expressed on immune cells, including neutrophils, monocytes, macrophages and activated T cells [5]–[7] The expression of uPAR is enhanced by inflammatory mediators such as bacterial phospholipases and pro-inflammatory cytokines [8]–[14]. Various soluble forms of uPAR (suPAR) have been found in body fluids and are increased by infectious diseases. [15]–[24]. High blood levels of suPAR has been shown to independently predict mortality in patients with various infections diseases including HIV, tuberculosis, malaria and sepsis [17], [20], [22], [25]–[27].

We have previously shown that suPAR is elevated in patients with active TB at treatment initiation and that suPAR levels decreased to normal levels after 8 months of TB treatment. Furthermore, we found that high suPAR at TB diagnosis was associated with increased mortality risk during the TB treatment period [17]. Finally, a South African study involving 20 HIV-negative, TB culture positive individuals found that suPAR was elevated in TB patients compared to 13 controls and that suPAR levels decreased significantly after one week of treatment and decreased to the level of controls at the end of treatment [28].

In this study, we measured suPAR at diagnosis, after 2 weeks, 1 month and 2, 5, and 8 months of treatment. The overall objective of the present study was to examine whether changes in suPAR during TB treatment can be used to monitor treatment efficacy.

## Study Populations and Methods

### Study Area

The study was conducted in an area included in the demographic surveillance system (DSS) of the Bandim Health Project (BHP) in Bissau, Guinea-Bissau. Individuals in the study area are registered with ID-number, age, gender, ethnic group and socio-economic factors. Censuses are performed at regular intervals and information on mortality and migration is collected.

### Study Population

All patients with symptoms or signs of TB were registered upon consultation at 3 health centers in the study area or admission to the National TB Hospital. Demographic information was recorded and blood (in EDTA tubes), urine and sputum samples were obtained. From April 2004 to December 2006, 1682 patients, older than 15 years of age, with respiratory symptoms were screened for TB. Among these, 466 (28%) were diagnosed as having active TB according to WHO guidelines and were eligible for the present study [29].

Of 466 eligible TB patients, 65 (14%) were excluded for the following reason: Clinical examination was not performed in 33 patients, 21 patients refused to participate or lived outside the study area while 11 had no available suPAR measurement at any time point. A further 123 (31%) patients had no baseline suPAR sample taken at diagnosis due to administrative constraints at one of the inclusion sites.

These patients were excluded from the analysis since focus was on the change in suPAR relative to the baseline level. In total, 278 patients were included in the present analysis.

Historical and physical examinations were performed at enrolment. HIV pre- and post-test counselling was conducted. Antiretroviral treatment was not available in Bissau at the start of the study (became available in 2007). HIV positive patients were referred to CIDA/ALTERNAG (voluntary counselling and testing centre) for bactrim treatment and counselling free of charge.

### TB Diagnosis

Patients presenting with a cough of more than 2–3 weeks duration were classified as pulmonary tuberculosis (PTB) suspects. These were asked to submit three sputum samples for acid-fast bacilli (AFB) microscopy following guidelines from the National Tuberculosis Programme (NTP). Sputum samples were examined using Ziehl-Nielsen stain. Patients with one or more AFB smear-positive samples were diagnosed as smear-positive PTB without further investigation. Smear-negative patients were given non-tuberculosis antibiotics for 2 weeks. Those who failed to improve were referred for further examination. The patient was diagnosed as smear-negative PTB if the clinical signs and X-ray findings were compatible with TB. The group of smear-positive and smear-negative PTB patients constituted our study population.

### Treatment Protocol

Patients were treated according to standard guidelines of the NTP consisting of an intensive phase with 2 months of Daily Observed Treatment (DOT) with Ethambutol (E), Isoniazid (H), Rifampicin (R) and Pyrazinamide (Z) followed by a continuation phase of 6 months of Isoniazide and Rifampicin. HIV infected and non-infected received the same treatment.

### Follow-up

Patients were visited at 2, 5′ and 8 month by a field assistant and were invited for clinical examination at the health centers according to the monitoring treatment guidelines provided by the NTP. Information on socio-economic and demographic characteristics, medical history and physical examination were recorded and blood samples were collected. If patients did not appear at the scheduled follow up visit, they were visited again and asked to attend the health centre. Additional sputum, urine, and blood samples were obtained at 2 weeks, and 1 month after treatment start.

### Laboratory Methods

Venous blood samples (5.0 ml) were collected in a microtainer evacuated blood collection system with additives (K2EK_2_EDTA – Greiner bio-one, USA). Blood samples were stored at −20°C at the National Laboratory. Plasma suPAR levels were determined in Guinea-Bissau using the suPARnostic® ELISA kit (ViroGates, Copenhagen, Denmark). The measured intra- and inter-assay variation was 6% and 9%, respectively.

HIV testing was performed in all TB patients with available plasma. Blood samples were analysed at the National Laboratory. Sera were screened using the Determine ™ HIV-1/2 Serum/Plasma Assay (Abbott, List N0. 7D23-13), and reactive sera were confirmed with both the Capillus HIV-1/HIV-2 (Cambridge Diagnostics, Galway, Ireland) and the ImmunoComb^R^ II HIV 1&2 BiSpot Anti HIV 1&2 EIA (Orgenics Ltd, Yavne, Israel). No HIV positive patients received antiretroviral therapy during the TB treatment period.

### Statistical Methods

Data were double entered in a Dbase V database and statistical analyses were conducted in STATA version 10. A level of 5% was used for significance. Geometric means were used to present average suPAR levels and p-values were obtained from t-tests of log-suPAR. Follow-up time was censored at 8 months in the survival analysis. Two Cox models were fitted. The covariates: inclusion suPAR, HIV status, gender and age were included in both models. The second model furthermore included a variable indicating whether suPAR increased or not between baseline and 1 month. A prognostic index ( = β·x) was calculated from both models and the area (AUC) under the receiver operator characteristic (ROC) curve was presented.

### Ethical Considerations

All patients provided written consent (or fingerprint) for giving blood and for the use of data for the present study. The Guinea-Bissau Government Ethics Committee and the European Union FP6 Scientific Ethics Committee approved the study protocol, N^a^. Ref^a^ No 011/DHE/2004, and LSSP-CT-2005-012173, respectively. Patients were not initially asked for an HIV test given the fact that there was no HIV treatment available at the time and great stigmatisation of HIV infected. Permission to retrospectively test samples for HIV-1 and HIV-2 was obtained from the Guinea-Bissau National Ethics Committee dated 02, December 2005. When antiretroviral treatment (ART) treatment became available in 2007, HIV positive assumed TB negative individuals were re-invited for clinical examination. At this examination, HIV testing was carried out and patients were offered ART based on immunological status (CD4 counts).

## Results

Baseline characteristics are shown in Table 1. Of the 278 included TB positive individuals, 176 were males (63%) and 102 females (37%). The median age was 33 years of age. There were 208 (75%) patients having smear-positive pulmonary TB and 70 (25%) with smear-negative pulmonary TB. HIV-status was unavailable for 25 (9%) patients. Among the remaining 253 patients, 65 (23%) were HIV-1 infected, including HIV-1+2 dual infections, and 27 (10%) were HIV-2 infected.

**Table 1:** Baseline characteristics and mortality according to quartiles of inclusion suPAR.

Characteristic	1^st^ suPAR Quartile(1.7–4.3)		2^nd^ suPAR Quartile(4.3–5.9)		3^rd^ suPAR Quartile(5.9–8.4)		4^th^ suPAR Quartile(8.4–54)		Total Range(1.7–54)		p-value
No. of subjects	69		70		69		70		278		
Median Age years*	34	(20–60)	32	(18–63)	33	(19–60)	32	(21–62)	33	(19–61)	0.66
Male	40	(58%)	45	(64%)	45	(65%)	46	(66%)	176	(63%)	0.76
Female	29	(42%)	25	(36%)	24	(35%)	24	(34%)	102	(37%)	
HIV-1	12	(17%)	9	(13%)	20	(29%)	24	(34%)	65	(23%)	<0.001
HIV-2	6	(9%)	8	(11%)	9	(13%)	4	(6%)	27	(10%)	
HIV negatives	49	(71%)	49	(70%)	37	(54%)	26	(37%)	161	(58%)	
Missing	2	(3%)	4	(6%)	3	(4%)	16	(23%)	25	(9%)	
Smear-positive	41	(59%)	58	(83%)	53	(77%)	56	(80%)	208	(75%)	0.007
Smear-negative	28	(41%)	12	(17%)	16	(23%)	14	(20%)	70	(25%)	

* Median and 5%–95% percentiles. P-value calculated by Kruskal-Wallis test.

During the 8-month treatment period, 31 (11%) patients died. In the smear-positive group, mortality was 24% for HIV-positive individuals (15/63), 2% (2/127) for HIV-negative individuals and 39% (7/18) for patients with unknown HIV status (Table 2). In the smear-negative group, mortality was 7% for HIV-positive individuals (2/29), 15% (5/34) for HIV-negative individuals and 0% (0/7) for patients with unknown HIV status.

**Table 2:** Mortality effect of high inclusion suPAR according to HIV and smear status.

	Mortality rate per 100 person-years(deaths/person-years of follow-up) [N]		Mortality rate Ratio^1^	Mortality rate Ratio^2^
	suPAR quartile 1–3(1.7–8.4 ng/ml)	suPAR quartile 4(8.4–54.0 ng/ml)		
**Smear Positive & Smear Negative**				
HIV negative	6.8 (6/88.0) [135]	5.8 (1/17.1) [26]	0.86 (0.10–7.12)	0.00 (−)
HIV positive	20.0 (8/40.1) [64]	57.5 (9/15.7) [28]	2.85 (1.10–7.40)	4.46 (1.27–15.6)
HIV status missing	17.3 (1/5.8) [9]	74.9 (6/8.0) [16]	4.29 (0.52–35.7)	3.78 (0.22–64.1)
Overall	11.2 (15/134.1) [208]	39.2 (16/40.8) [70]	3.47 (1.72–7.03)	2.84 (1.17–6.89)
**Smear Positive only**				
HIV negative	1.4 (1/69.2) [104]	6.6 (1/15.1) [23]	4.55 (0.28–72.8)	0.00 (−)
HIV positive	26.9 (7/26.1) [43]	77.5 (8/10.3) [20]	2.81 (1.02–7.77)	5.44 (0.99–29.8)
HIV status missing	32.0 (1/3.1) [5]	100.0 (6/6.0) [13]	3.08 (0.37–25.6)	1.56 (0.08–30.4)
Overall	9.2 (9/98.4) [152]	47.7 (15/31.4) [56]	5.09 (2.23–11.7)	3.71 (1.25–11.1)

1 Using time since treatment initiation as underlying time.

2 Using age as underlying time.

Among the 188 excluded individuals, 111 (59%) were males and 77 (41%) females. The median age was 34 years of age. HIV-status was unavailable for 39 (21%) patients. There were 35 (19%) HIV-1 infected individuals, including dual infections, and 9 (5%) individuals were HIV-2 infected.

### Smear Conversion and Mortality

There were 208 smear-positive patients included. Two patients died one day after treatment initiation. Sputum smear samples at 1 month were available for 163 (79%) of the remaining 206 subjects. Smear conversion was observed in 107/163 (66%) patients with a mean inclusion suPAR of 5.85 ng/ml and a subsequent mortality of 8% (9/107) between 1 and 8 months of treatment. Among the 56 patients who had not smear converted at 1 month, the mean inclusion suPAR level was 6.24 ng/ml and the subsequent mortality was 7% (4/56) The difference in suPAR at time of diagnosis was not significantly different between those who did or did not smear convert after 1 month of treatment (p = 0.41).

At 2 months, one more patient had died leaving 205 subjects at risk. Among the 153 (75%) patients with an available 2 months sputum sample, smear conversion was observed in 141 (92%) individuals with a subsequent mortality of 5% (7/141) between 2 and 8 months of treatment. No deaths were observed among the 12 patients continuing to be smear-positive. The mean suPAR level at diagnosis was 5.87 ng/ml among individuals with 2-month smear conversion compared to a mean suPAR level of 7.36 ng/ml in the 12 individuals without smear conversion. This difference was not significant (p = 0.10).

### SuPAR as a Predictor of Subsequent Mortality

The inclusion suPAR level was strongly associated with mortality during treatment. Mortality was especially high in the 4^th^ quartile (suPAR>8.4 ng/ml) with an observed mortality of 23% (16/70) during treatment compared to a mortality of 7% (15/208) in the other three quartiles (p = 0.004). The resulting risk ratio (RR) of being in the highest suPAR quartile was 3.1 (95% CI: 1.65–6.07). Regarding the suPAR level at 1 month, Individuals in the highest quartile had a RR of 2.16 (95% CI: 0.91–5.13) compared to the three lowest quartiles. At 2 months, the RR was 2.07 (95% CI: 0.82–5.21). As shown in table 2, the high mortality risk among patients in the highest suPAR quartile was confined to TB patients with concurrent HIV infection.

Among the 278 included patients, 190 had additional samples taken at 2 weeks, 174 had samples obtained at 1 month and 186 had samples obtained at 2 months. Mortality following inclusion was high among patients that did not return to the clinic for follow-up samples, with a mortality rate of 0.25/per person year of observation (PYO) (Table 2). The increased mortality associated with missing samples following inclusion was mainly observed for individuals with a high suPAR level at inclusion.

### Increase in suPAR during Treatment and Risk of Mortality

**Figure 1:**
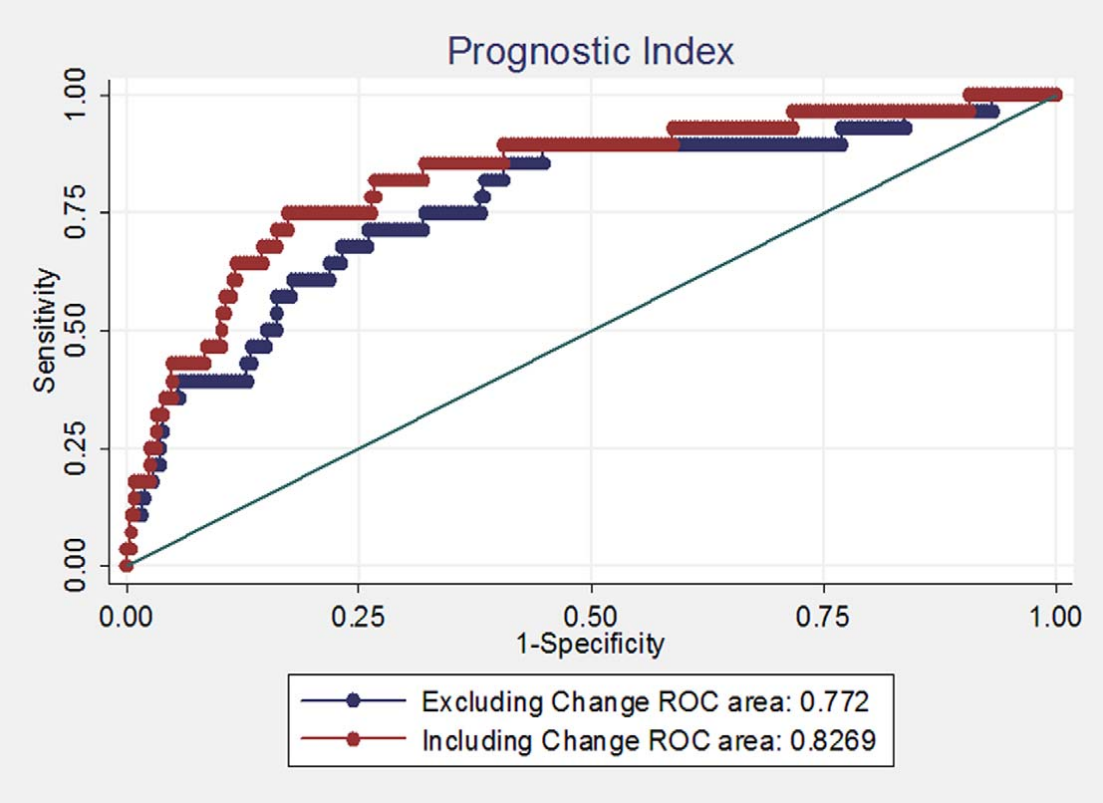
ROC-curves based on the prognostic index of inclusion suPAR, HIV status, gender, age and with (red line) or without (blue line) increased suPAR level at 1 month included in the model.

Table 3 explores the effect on mortality of increased suPAR following treatment. After 2 weeks of treatment no significant effect of increased suPAR was observed; compared to patients with declined suPAR, the Mortality Rate Ratio (MRR) was 0.93 (0.28–3.06). At 1 month, a 4-fold higher mortality rate was observed among individuals with increased suPAR level compared to individuals with decreased suPAR (MRR = 4.53 (1.45–14.1)) and at 2 months the mortality rate was doubled (MRR = 2.05 (0.62–6.82)). After 5 months, only one patient died and thus no analysis was carried out. AUC for the prognostic index based on inclusion suPAR, HIV-status, gender and age was 0.77 (Figure 1, blue line). When including whether or not the patient’s suPAR level increased after 1-month of treatment, the AUC increased to 0.83 (Figure 1, red line).

**Table 3:** Mortality effect of increased suPAR stratified by quartiles of inclusion suPAR.

Inclusion SuPAR Quartile	Increased suPAR	2 weeks Rate (Died/PYO) [N]	1 month Rate (Died/PYO) [N]	2 months Rate (Died/PYO) [N]
Quartile 1–3	NoYesMissing	13.0 (6/46) [76]7.7 (3/39) [63]14.6 (6/41) [69]	7.3 (3/41) [71]17.6 (6/34) [61]11.9 (5/42) [76]	10.2 (6/59) [123]13.3 (2/15) [31]12.5 (3/24) [54]
4th quartile	NoYesMissing	23.1 (6/26) [45]50.0 (1/2) [4]70.0 (7/10) [21]	11.1 (2/18) [33]100.0 (3/3) [7]64.3 (9/14) [33]	20.0 (2/10) [22]2/2 (0.89) [6]43.7 (7/16) [42]
Total	NoYesMissing	16.7 (12/72) [121]4/41 (0.10) [67]13/52 (0.25) [90]	8.5 (5/59) [104]24.3 (9/37) [68]25.0 (14/56) [106]	11.6 (8/69) [145]23.5 (4/17) [37]25.0 (10/40) [96]
Rate Ratio*	NoYesMissing	10.93 (0.28–3.06)1.82 (0.82–4.04)	1**4.53 (1.45–14.1)** **3.19 (1.15–8.87)**	12.05 (0.62–6.82)1.51 (0.57–4.01)

* Adjusted for the highest inclusion quartile.

Rate (Died/PYO) [N] = % died (died/Person Year of Observation) [Number of individuals]. Significant Rate Ratios are shown in **bold.**

## Discussion

Markers of TB treatment efficacy may aid in identification of patients at continued risk of a negative outcome. Smear conversion is often used to assess treatment efficacy among smear-positive pulmonary TB patients. There were 208 smear-positive TB patients in our cohort. After 1 month of treatment, 66% were observed to be smear-negative and nearly all (92%) were smear-negative after 2 months of treatment. Our results showed no association between early smear conversion and subsequent mortality nor were smear conversion related to significantly lower inclusion suPAR. We therefore focused on mortality as marker of treatment failure.

Of all the individuals suspected of having TB, only 25% is found to be TB positive. Among the 75% not found to be TB positive, we have recently shown that high suPAR is associated with high risk of dying within 3 months [29]. The present results extend this finding to show that suPAR is prognostic for all patients regardless of a TB diagnosis. We recently reported that suPAR is prognostic for disease and mortality in the general Danish population [30]. Thus, an elevated suPAR level is associated with increased risk of a negative outcome irrespective of diagnosis. Whether the suPAR level reflects disease processes or plays a direct role in the pathogenesis is unknown. A resent study showed that suPAR is a direct cause of at least one disease, namely glomerulosclerosis [31].

The current study included measurements of suPAR during treatment. We expected that an increase in suPAR after treatment initiation would be associated with a higher mortality risk. We did observe a significant effect of increased suPAR after 1 month of treatment, which may indicate treatment failure. However, resistance-testing facilities were not available during the study. Thus, further studies are needed to determine whether there is an association between increases in suPAR after 1-month of treatment and resistance development.

The Cox regression survival model showed that most of the elevated mortality risk could be predicted by inclusion suPAR followed by HIV-status and age. In a prognostic index based on inclusion suPAR, HIV-status, gender and age, the change in suPAR after one month of treatment added some further value to the model, as illustrated in the ROC curve (Figure 1).

The prevalence of HIV was high in the present cohort of TB patients. Among those tested, 32% were HIV-infected. Mortality during treatment was especially high in the HIV-infected group and the group that did not come for further follow-up visits. We suspect that many in the latter group actually were HIV positive. The two groups together accounted for 77% (24) of the 31 deaths observed. The high prevalence of HIV among TB patients shows the importance of routine HIV testing and of enrolling these patients into HIV treatment programs.

A high suPAR level at inclusion or at any point during treatment is associated with increased mortality. Patients in these groups should therefore be monitored closely. An important practical issue, that needs to be addressed, is how to define what a high suPAR level is. As the risk of mortality increases linearly with raised suPAR levels, it depends on the capacity for further clinical and laboratory examination. We observed that missing or increased suPAR at 1 or 2 months was associated with increased mortality risk. However, the effects of inclusion suPAR and HIV-status were stronger.

How could the knowledge of baseline suPAR and changes in suPAR influence on clinical practise in a resource restrained setting such as in Guinea-Bissau? A high or increasing suPAR level should lead to further clinical and laboratory investigations. Do the TB patient have comorbidities, which leads to elevated suPAR levels [32], that could explain the increased risk of mortality? Testing for TB resistance, HIV-coinfection, pneumonia, malaria and non-infectious diseases such as cancer or type 2 diabetes can lead to initiation or changes in treatment that may decrease risk of mortality. However, further clinical and laboratory testing will put additional pressure on the already resource restrained health system. In this and previous studies on suPAR, it is evident that patients with low suPAR (e.g. below 3.5 ng/ml) have low risk of mortality; Perhaps reduction in clinical monitoring (e.g. fewer clinical examinations during the treatment period), or less laboratory testing (e.g. no resistance or co-infection testing) could reallocate resources to those with high suPAR in need of further testing. We are currently conducting a prospective study to optimize an algorithm combining a clinical TB score [33] with the suPAR value. When an optimal prognostic algorithm has been developed, this should be properly assessed in randomized trial testing whether this algorithm can reduce mortality. As cost is always an issue, the randomized study may take into account reallocation of resources from low-risk to high-risk individuals.

Missing baseline suPAR measurements were a major limitation of the present study. For 31% of the 401 included patients no sample was taken before treatment. Many of the TB patients went for their first consultancy at the reference TB Hospital. Administrative constraints at the TB hospital restricted our collection of blood samples at treatment start.

In conclusion, a high suPAR level at inclusion was associated with an increased mortality risk. The change in suPAR after 14 days of treatment was not associated with subsequent risk, while an increase in suPAR after one month of treatment was associated with increased risk of dying during the remaining treatment period.

## References

[1] Corbett EL, Watt CJ, Walker N, Maher D, Williams BG, et al (2003) The growing burden of tuberculosis: global trends and interactions with the HIV epidemic. Arch Intern Med 163(9): 1009–1021. 12742798 10.1001/archinte.163.9.1009

[2] Dye C, Scheele S, Dolin P, Pathania V, Raviglione MC (1999) Consensus statement. Global burden of tuberculosis: estimated incidence, prevalence, and mortality by country. WHO Global Surveillance and Monitoring Project. JAMA 282(7): 677–686. 10517722 10.1001/jama.282.7.677

[3] WHO (2004). Global Tuberculosis Control. Surveillance, Planning and Financing. WHO Report. Geneva, Switzerland World Health Organization: 1–218.

[4] Gustafson P, Gomes VF, Vieira CS, Rabna P, Seng R, et al (2004) Tuberculosis in Bissau: incidence and risk factors in an urban community in sub-Saharan Africa. Int J Epidemiol 33(1): 163–172. 15075165 10.1093/ije/dyh026

[5] Plesner T, Behrendt N, Ploug M (1997) Structure, function and expression on blood and bone marrow cells of the urokinase-type plasminogen activator receptor, uPAR. Stem Cells 15(6): 398–408. 9402652 10.1002/stem.150398

[6] Ploug M (2003) Structure-function relationships in the interaction between the urokinase-type plasminogen activator and its receptor. Curr Pharm Des 9(19): 1499–1528. 12871065 10.2174/1381612033454630

[7] Nykjaer A, Moller B, Todd RF 3rd, Christensen T, Andreasen PA, et al (1994) Urokinase receptor. An activation antigen in human T lymphocytes. J Immunol 152(2): 505–516. 8283034

[8] Plesner T, Ralfkiaer E, Wittrup M, Johnsen H, Pyke C, et al (1994) Expression of the receptor for urokinase-type plasminogen activator in normal and neoplastic blood cells and hematopoietic tissue. Am J Clin Pathol 102(6): 835–841. 7801901 10.1093/ajcp/102.6.835

[9] Coleman JL, Gebbia JA, Benach JL (2001) Borrelia burgdorferi and other bacterial products induce expression and release of the urokinase receptor (CD87). J Immunol 166(1): 473–480. 11123326 10.4049/jimmunol.166.1.473

[10] Coleman JL, Benach JL (2003) The urokinase receptor can be induced by Borrelia burgdorferi through receptors of the innate immune system. Infect Immun 71(10): 5556–5564. 14500474 10.1128/IAI.71.10.5556-5564.2003 PMC201106

[11] Dekkers PE, ten Hove T, te Velde AA, van Deventer SJ, van Der Poll T (2000) Upregulation of monocyte urokinase plasminogen activator receptor during human endotoxemia. Infect Immun 68(4): 2156–2160. 10722614 10.1128/iai.68.4.2156-2160.2000 PMC97398

[12] Juffermans NP, Dekkers PE, Verbon A, Speelman P, van Deventer SJ, et al (2001) Concurrent upregulation of urokinase plasminogen activator receptor and CD11b during tuberculosis and experimental endotoxemia. Infect Immun 69(8): 5182–5185. 11447203 10.1128/IAI.69.8.5182-5185.2001 PMC98617

[13] Kirchheimer JC, Nong YH, Remold HG (1998) IFN-gamma, tumor necrosis factor-alpha, and urokinase regulate the expression of urokinase receptors on human monocytes. J Immunol 141(12): 4229–4234. 2848891

[14] Sitrin RG, Todd RF, 3rd, Mizukami IF, Gross TJ, Shollenberger SB, et al (1994) Cytokine-specific regulation of urokinase receptor (CD87) expression by U937 mononuclear phagocytes. Blood 84(4): 1268–1275. 8049441

[15] Alfano M, Sidenius N, Blasi F, Poli G (2003) The role of urokinase-type plasminogen activator (uPA)/uPA receptor in HIV-1 infection. J Leukoc Biol 74(5): 750–756. 12960238 10.1189/jlb.0403176

[16] de Bock CE, Wang Y (2004) Clinical significance of urokinase-type plasminogen activator receptor (uPAR) expression in cancer. Med Res Rev 24(1): 13–39. 14595671 10.1002/med.10054

[17] Eugen-Olsen J, Gustafson P, Sidenius N, Fischer TK, Parner J, et al (2002) The serum level of soluble urokinase receptor is elevated in tuberculosis patients and predicts mortality during treatment: a community study from Guinea-Bissau. Int J Tuberc Lung Dis 6(8): 686–692. 12150480

[18] Florquin S, van den Berg JG, Olszyna DP, Claessen N, Opal SM (2001) Release of urokinase plasminogen activator receptor during urosepsis and endotoxemia. Kidney Int 59(6): 2054–2061. 11380806 10.1046/j.1523-1755.2001.00719.x

[19] Ostrowski SR, Piironen T, Hoyer-Hansen G, Gerstoft J, Pedersen BK, et al (2005) Reduced release of intact and cleaved urokinase receptor in stimulated whole-blood cultures from human immunodeficiency virus-1-infected patients. Scand J Immunol 61(4): 347–356. 15853918 10.1111/j.1365-3083.2005.01582.x

[20] Ostrowski SR, Ullum H, Goka BQ, Hoyer-Hansen G, Obeng-Adjei G, et al (2005) Plasma concentrations of soluble urokinase-type plasminogen activator receptor are increased in patients with malaria and are associated with a poor clinical or a fatal outcome. J Infect Dis 191(8): 1331–1341. 15776381 10.1086/428854

[21] Perch M, Kofoed P, Fischer TK, Co F, Rombo L, et al (2004) Serum levels of soluble urokinase plasminogen activator receptor is associated with parasitemia in children with acute Plasmodium falciparum malaria infection. Parasite Immunol 26(5): 207–211. 15491469 10.1111/j.0141-9838.2004.00695.x

[22] Sidenius N, Sier CF, Ullum H, Pedersen BK, Lepri AC, et al (2000) Serum level of soluble urokinase-type plasminogen activator receptor is a strong and independent predictor of survival in human immunodeficiency virus infection. Blood 96(13): 4091–4095. 11110678

[23] Slot O, Brunner N, Locht H, Oxholm P, Stephens RW (1999) Soluble urokinase plasminogen activator receptor in plasma of patients with inflammatory rheumatic disorders: increased concentrations in rheumatoid arthritis. Ann Rheum Dis 58(8): 488–492. 10419867 10.1136/ard.58.8.488 PMC1752924

[24] Wittenhagen P, Kronborg G, Weis N, Nielsen H, Obel N, et al (2004) The plasma level of soluble urokinase receptor is elevated in patients with Streptococcus pneumoniae bacteraemia and predicts mortality. Clin Microbiol Infect 10(5): 409–415. 15113317 10.1111/j.1469-0691.2004.00850.x

[25] Lawn SD, Myer L, Bangani N, Vogt M, Wood R (2007) Plasma levels of soluble urokinase-type plasminogen activator receptor (suPAR) and early mortality risk among patients enrolling for antiretroviral treatment in South Africa. BMC Infect Dis 7: 41. 17509133 10.1186/1471-2334-7-41 PMC1885800

[26] Huttunen R, Syrjänen J, Vuento R, Hurme M, Huhtala H, et al (2011) Plasma level of soluble urokinase-type plasminogen activator receptor as a predictor of disease severity and case fatality in patients with bacteraemia: a prospective cohort study. J Intern Med 270(1): 32–40. 21332843 10.1111/j.1365-2796.2011.02363.x

[27] Koch A, Voigt S, Kruschinski C, Sanson E, Dückers H, et al (2011) Circulating soluble urokinase plasminogen activator receptor is stably elevated during the first week of treatment in the intensive care unit and predicts mortality in critically ill patients. Crit Care 15(1): R63. 21324198 10.1186/cc10037 PMC3221996

[28] Djoba Siawaya JF, Bapela NB, Ronacher K, Veenstra H, Kidd M, et al (2008) Immune parameters markers of tuberculosis extent of disease and early prediction of anti-tuberculosis chemotherapy response. Journal of Infection 56(5): 340–7. 18359089 10.1016/j.jinf.2008.02.007

[29] Rabna P, Andersen A, Wejse C, Oliveira I, Gomes VF, et al (2009) High mortality risk among individuals assumed to be TB-negative can be predicted using a simple test. Trop Med Int Health 14: 986–994. 19725925 10.1111/j.1365-3156.2009.02328.x

[30] Eugen-Olsen J, Andersen O, Linneberg A, Ladelund S, Hansen TW, et al (2010) Circulating soluble urokinase plasminogen activator receptor predicts cancer, cardiovascular disease, diabetes and mortality in the general population. J Intern Med 268(3): 296–308. 20561148 10.1111/j.1365-2796.2010.02252.x

[31] Wei C, El Hindi S, Li J, Fornoni A, Goes N, et al (2011) Circulating urokinase receptor as a cause of focal segmental glomerulosclerosis. Nat Med17(8): 52–60. 10.1038/nm.2411 PMC4089394 21804539

[32] Haupt TH, Petersen J, Ellekilde G, Klausen HH, Thorball CW, et al (2012) suPar levels are associated with mortality, admission time, and Charlson Comorbidity Index in the acutely admitted medical patient: a prospective observational study. Crit Care 16: R130. 22824423 10.1186/cc11434 PMC3580714

[33] Wejse C, Gustafson P, Nielsen J, Gomes VF, Aaby P, et al (2008) TBscore: Signs and symptoms from tuberculosis patients in a low-resource setting have predictive value and may be used to assess clinical course. Scand J Infect Dis 40(2): 111–20. 17852907 10.1080/00365540701558698

